# Metabolic Reprogramming of *Clostridioides difficile* During the Stationary Phase With the Induction of Toxin Production

**DOI:** 10.3389/fmicb.2018.01970

**Published:** 2018-08-21

**Authors:** Julia D. Hofmann, Andreas Otto, Mareike Berges, Rebekka Biedendieck, Annika-Marisa Michel, Dörte Becher, Dieter Jahn, Meina Neumann-Schaal

**Affiliations:** ^1^Department of Bioinformatics and Biochemistry, Technische Universität Braunschweig, Braunschweig, Germany; ^2^Braunschweig Integrated Centre of Systems Biology (BRICS), Braunschweig, Germany; ^3^Department for Microbial Proteomics, University of Greifswald, Greifswald, Germany; ^4^Institute of Microbiology, Technische Universität Braunschweig, Braunschweig, Germany; ^5^Leibniz Institute DSMZ – German Collection of Microorganisms and Cell Cultures, Braunschweig, Germany

**Keywords:** *Clostridium difficile*, *Clostridioides difficile*, metabolism, toxin formation, starvation, Stickland reactions

## Abstract

The obligate anaerobe, spore forming bacterium *Clostridioides difficile* (formerly *Clostridium difficile*) causes nosocomial and community acquired diarrhea often associated with antibiotic therapy. Major virulence factors of the bacterium are the two large clostridial toxins TcdA and TcdB. The production of both toxins was found strongly connected to the metabolism and the nutritional status of the growth environment. Here, we systematically investigated the changes of the gene regulatory, proteomic and metabolic networks of *C. difficile* 630Δ*erm* underlying the adaptation to the non-growing state in the stationary phase. Integrated data from time-resolved transcriptome, proteome and metabolome investigations performed under defined growth conditions uncovered multiple adaptation strategies. Overall changes in the cellular processes included the downregulation of ribosome production, lipid metabolism, cold shock proteins, spermine biosynthesis, and glycolysis and in the later stages of riboflavin and coenzyme A (CoA) biosynthesis. In contrast, different chaperones, several fermentation pathways, and cysteine, serine, and pantothenate biosynthesis were found upregulated. Focusing on the Stickland amino acid fermentation and the central carbon metabolism, we discovered the ability of *C. difficile* to replenish its favored amino acid cysteine by a pathway starting from the glycolytic 3-phosphoglycerate via L-serine as intermediate. Following the growth course, the reductive equivalent pathways used were sequentially shifted from proline via leucine/phenylalanine to the central carbon metabolism first to butanoate fermentation and then further to lactate fermentation. The toxin production was found correlated mainly to fluxes of the central carbon metabolism. Toxin formation in the supernatant was detected when the flux changed from butanoate to lactate synthesis in the late stationary phase. The holistic view derived from the combination of transcriptome, proteome and metabolome data allowed us to uncover the major metabolic strategies that are used by the clostridial cells to maintain its cellular homeostasis and ensure survival under starvation conditions.

## Introduction

*Clostridioides difficile* (formerly *Clostridium difficile*) is an obligate anaerobe, spore forming, Gram-positive bacterium. Currently, it is the major cause of nosocomial and community acquired diarrhea, often associated with antibiotic therapy (Lessa et al., 2015). The symptoms vary from mild diarrhea to pseudomembranous colitis. In severe cases, the disease leads to acute inflammation of the colon and to life-threatening loss of fluids. In the last few years the pathogen has become a major threat to hospitalized patients (Dong et al., 2008) due to the occurrence of hyper-virulent strains and observed increasing antibiotic resistances (Loo et al., 2005; Warny et al., 2005; Baines et al., 2008; Stabler et al., 2009; Spigaglia et al., 2011; Tenover et al., 2012).

The major virulence factors of the bacterium are the two large clostridial toxins A (TcdA) and B (TcdB), an enterotoxin and a cytotoxin, which are responsible for the major symptoms of the disease. During *C. difficile in vitro* cultivations toxin formation and excretion was primarily detectable in the stationary phase. Only small amounts of toxins were detected intracellularly (Karlsson et al., 2008; Neumann-Schaal et al., 2015). Toxin A and B operate as glycosyltransferases to modify small GTP-binding proteins of target cells. This leads to a disruption of the actin cytoskeleton of intestinal epithelium cells (Just et al., 1995; Eichel-Streiber et al., 1996). The corresponding genes *tcdA* and *tcdB* as well as the regulatory genes *tcdR, tcdE*, and *tcdC* are located at a single chromosomal site called the pathogenicity locus (PaLoc) (Mani and Dupuy, 2001; Carter et al., 2011; Govind and Dupuy, 2012). Apart from the main virulence factors, high virulent strains additionally have the capacity to produce a third so called binary toxin named CDT, but so far the overall role of CDT is not completely understood (Warny et al., 2005; Hemmasi et al., 2015).

For clostridial growth and toxin mediated pathogenicity, the metabolic network and the nutritional status in the environment play an important role. The presence of the amino acids cysteine and proline reduces toxin production in dependence of the used growth medium and tested strain (Karlsson et al., 2000). Dubois et al. (2016) suggested that the cysteine-dependent toxin gene regulation is responding to products of the cysteine degradation, mainly pyruvate and probably sulfide. Analogously, addition of a mixture of the seven amino acids glycine, isoleucine, leucine, methionine, threonine, tryptophan, and valine as well as biotin to growing *C. difficile* leads to similar observations (Karlsson et al., 1999, 2000, 2008). Carbohydrates also affect toxin production. In complex medium glucose or other rapidly metabolizable sugars lead to reduced toxin production (Dupuy and Sonenshein, 1998; Karlsson et al., 1999). Interestingly, in minimal media, the supplementation of glucose increases toxin formation (Karlsson et al., 1999). The background of this discrepancy is currently not completely known.

A major process for energy production by *C. difficile* is the Stickland reaction, a coupled fermentation of amino acids (Stickland, 1934). After initial enzymatic deamination, the 2-ketoacids are either oxidized or reduced in a coupled reaction to their corresponding organic acids. Energy is conserved by substrate-level phosphorylation mainly in the oxidative. The resulting electrons are assimilated in the reductive path. The reductive product is a carboxylic acid with the same length as the original amino acid. The oxidative product is a carboxylic acid one atom shorter than the original amino acid. Depending on the amino acid, one amino acid is oxidized while up to two are reduced. Certain amino acids like proline and glycine are processed via modified Stickland pathways (Stadtman, 1966; Jackson et al., 2006).

Besides the Stickland reactions, classical fermentation pathways are important alternatives for *C. difficile* to gain energy. In addition to glucose, central carbon metabolism associated amino acids are degraded. Alanine, cysteine, and serine are introduced into the central metabolism via pyruvate. Threonine is degraded via acetaldehyde and glycine to acetyl-CoA, or via 2-oxobutanoate to propanoyl-CoA (Fonknechten et al., 2010). The main products of the butanoate fermentation are butanoate and pentanoate. Other fermentation products of the central carbon metabolism are propanoate, lactate, acetate and ethanol (Aboulnaga et al., 2013; Dannheim et al., 2017a).

In addition to substrate level and electron transport phosphorylation the proton-translocating ferredoxin:NAD oxidoreductase RNF complex catalyzes a process called flavin-based electron bifurcation which can be regarded as a third mode of energy conservation (Aboulnaga et al., 2013; Buckel and Thauer, 2013). The RNF complex couples the oxidation of a ferredoxin to the reduction of NAD^+^ with ion (H^+^ or Na^+^) pumping across the cytoplasmic membrane. The resulting ion gradient can be used for ATP synthesis (Biegel et al., 2011; Tremblay et al., 2012; **Supplementary Figure S1**). The reduced ferredoxin and NAD^+^ are typical products of the *C. difficile* fermentation metabolism (**Supplementary Figure S1**). In contrast to the metabolism employed for growth, there is only limited knowledge about the metabolic rearrangements during the transient and stationary growth phase at the onset of toxin production of *C. difficile*. However, the later phases harbor the onset of toxin production in *C. difficile*.

The integrated view resulting from the combination of transcriptome, proteome and metabolome data allows to identify major cellular strategies during an adaptation process. Here, we present the first time-resolved multi-omics approach to unravel the molecular basis of global changes taking place in *C. difficile* while shifting from a growing to a non-growing state. Changes in metabolism with involved pathways and enzymes in combination with underlying gene regulatory networks were our major target. Special attention was given to the production of the various virulence factors in this context.

## Materials and Methods

### Strain, Media and Growth Conditions

Studies were performed with *C. difficile* 630Δ*erm* (DSM28645) (Hussain et al., 2005) obtained from the German Collection of Microorganisms and Cell Cultures (DSMZ, Braunschweig, Germany).

Main cultures were cultivated in CDMM as described earlier (Neumann-Schaal et al., 2015). Casamino acids were obtained from Roth (Carl Roth GmbH, Karlsruhe, Germany).

Cells were transferred twice with a dilution of 1:1000 in CDMM prior to inoculation of the main culture. Main cultures were inoculated with a dilution of 1:400 at an OD_600__nm_ of ∼0.003. Growth experiments were performed at 37°C and samples were taken in exponential phase after 14.5 h (exp) of cultivation, in transient phase (17.25 h, trans) and three times in stationary phase (19.25 h: stat1, 24.25 h: stat2 and 29.25 h: stat3).

### Cell Harvest and Analysis of Intracellular Metabolites

For every time point four independent cultures were sampled. Samples for metabolome analysis, CoA measurements, transcriptomics and proteomics were harvested anaerobically by centrifugation (10 min, 10,000 rpm, 4°C) using gas-tight polypropylene tubes (TPP, Trasadingen, Switzerland). The precipitated cells for proteomics, transcriptomics and CoA measurements were immediately frozen in liquid nitrogen after centrifugation. The supernatant for proteomic and extracellular metabolome analysis was sterile-filtered and frozen at -80°C.

For the analysis of intracellular metabolites the supernatant was removed and the precipitated cells were immediately quenched in pre-cooled isotonic sodium chloride/methanol [50% (v/v), -32°C] by resuspension. Cells were centrifuged again (5 min, 10,000 rpm, -20°C) to remove the quenching solution. The precipitated cells were then frozen in liquid nitrogen.

To determine sporulation of the cells, cultures were split into two aliquots. One on them was heated to 65°C for 20 min. Aliquots were plated on BHIS (37 g/L BHI, 5 g/L yeast extract, 0.5 g/L cysteine, 15 g/L agar) for vegetative cells and BHIS + 0.1% taurocholic acid for the germination of spores in different dilutions. The plates were cultivated in an anaerobic jar up to 48 h at 37°C. The colonies were counted and compared to each other.

### Transcriptomic Analysis

#### RNA Extraction

Bacterial RNA was extracted using the Qiagen RNeasy Kit Mini (Qiagen, Hilden, Germany) according to the manufacturer’s instructions with minor modifications. Briefly, precipitated cells from a 45 mL culture were resolved in 200 μL TE buffer and 15 mg/mL lysozyme, incubated for 30 min at room temperature and vigorously mixed every 2 min for 10 s for enzymatically disruption of the cells. Approximately 700 μL of RLT buffer and one spatula of glass beads were added and mixed vigorously for 3 min for mechanical disruption of the cells. Samples were centrifuged (3 min, 10,000 rpm, 4°C) and the supernatant was mixed with 470 μL of 100% ethanol. Afterwards, RNA purification was carried out using the Qiagen RNeasy Kit protocol. DNA contaminating the RNA samples was removed using RNase-free DNase I twice (Qiagen, Hilden, Germany). The RNA was further assessed by analysis on the Bioanalyzer 2100 (Agilent, Santa Clara, CA, United States) and RNA 6000 Nano Reagents (Agilent, Santa Clara, CA, United States). According to the amount of RNA extracted and its quality [RNA integrity numbers (RINs) > 8], the best samples per time point were used for transcriptomic analysis.

#### Microarray Experiment and Data Analysis

A customized whole-genome DNA microarray (8 × 15K format; Agilent, Santa Clara, CA, United States) of the genes of *C. difficile* 630Δ*erm* was designed with the eArray platform from Agilent according to recently published revised genome sequence (Sebaihia et al., 2006; Dannheim et al., 2017a). One microgram of isolated total cellular RNA was labeled with either Cy3 or Cy5 with the ULS fluorescent labeling kit for Agilent arrays (Kreatech, Amsterdam, Netherlands) according to the manufacturer’s manual. Subsequently, 300 ng of each labeled RNA was pooled, fragmented, and hybridized according to the “two-color microarray” protocol from Agilent. The DNA microarrays were scanned with an Agilent C scanner. All slides were analyzed using the statistical software environment R^1^ with the cran package gplots (Wang et al., 2000) and the bioconductor packages LIMMA (Hwa and Yang, 2007; Ritchie et al., 2015). Background signals were subtracted using the normexp method (Ritchie et al., 2007). A convolution of normal and exponential distributions was fitted to the foreground intensities using the background intensities as a covariate. All the corrected intensities of Cy3 and Cy5 were positive. Within each array a Loess normalization was performed (Yang et al., 2002). This normalization fitted locally using repeatedly weighted least squares and resulted in equal distributed up- and downregulated genes with 20–30% differentially expressed genes. A quantile normalization between the arrays ensured that the intensity is equal distributed across all arrays (Yang and Thorne, 2003). A linear model was fitted for each comparison of interest. The obtained p-values were adjusted for false discovery rate (FDR) using the method by Benjamini and Hochberg (1995). A gene was considered differentially expressed when the *p*-value was < 0.05 and -1 < log2 FC > 1 in comparison with its expression during exponential phase. The complete experimental data sets were deposited in the GEO database with the accession number GSE115054.

### Proteome Analysis

#### Sample Preparation

Cytosolic and membrane (“insoluble”) samples for proteome analyses were treated as described before (Otto et al., 2016). Briefly, cells were disrupted by sonication and after discarding of the cell debris, the membrane fraction was separated from the soluble cytosolic proteins by ultracentrifugation. For the extracellular protein fraction, affinity-bead based enrichment was used (Bonn et al., 2014). Both, proteins from the extracellular and the membrane fraction were loaded on an SDS PAGE gel and separated according to denatured molecular weight of the proteins.

#### LC-MS/MS-Analysis of the Proteome Fractions

Each lane of the SDS PAGE gel containing proteins from the membrane fraction and the extracellular fraction were cut into 10 equidistant pieces and subjected to tryptic digestion as described previously (Bonn et al., 2014). The resulting tryptic peptide mixtures were subjected to LC-MS/MS analysis on a TripleTOF 5600 instrument (AB Sciex, Concord, Canada) coupled with an Easy nLC-1000 (Thermo Scientific, Waltham, MA, United States). Sample desalting and elution by a binary gradient of water/acetonitrile was performed on an in-house packed column. Overview scans with a resolution of *R* = 30,000 in the range of 350–1,250 m/z with a scan time of 250 ms were followed by 20 MS/MS fragment scans in the range of 300–1,600 m/z with a maximum scan time of 50 ms after collisionally induced dissociation (CID).

For analysis of the cytosolic proteins, proteins were reduced/alkylated and subjected to in solution tryptic digest (Junker et al., 2018). The resulting peptide mixture was subjected to LC-MS/MS analysis on a Velos Elite (Thermo Scientific) coupled with an Easy nLC-1000 (Thermo Scientific) as described in Otto et al. (2016).

#### Proteome Data Analysis

Sequence database searching and quantification based on LFQ was performed by MaxQuant (version 1.5.3.30) (Cox and Mann, 2008). Only fully tryptic peptides with a maximum of two missed cleavages were allowed. Database searching was based on the fasta sequences for *C. difficile* 630Δ*erm* (Dannheim et al., 2017a) and a set of common contaminant proteins.

For cytosolic samples, methionine oxidation and cysteine carbamidomethylation was considered as variable modification, omitting the latter for the extracellular and the membrane fraction. Peptide and protein identifications were further processed with a false discovery rate (FDR) of less or equal 1%. For protein identification, a minimum of at least two unique peptides was set as threshold. Quantification was based on LFQ values obtained from the MaxQuant analysis. The data were processed within Perseus (1.5.3.3).

The mass spectrometry proteomics data have been deposited to the ProteomeXchange Consortium via the PRIDE partner repository (Vizcaíno et al., 2016) with the identifier PXD010498.

### Metabolic Analyses

#### Gas Chromatography/Mass Spectrometry Based Analysis of Intracellular and Extracellular Compounds

For extraction, the precipitated cells were re-suspended in 1.5 mL methanol containing 0.4 μg/mL ribitol per 10 mg cell dry weight. After incubation in an ultrasonic bath for 15 min at 70°C and cooling on ice, the same volume of water was added. After mixing (1 min, 2,000 rpm), chloroform was added in a volume of 2/3 of that of methanol. After a second mixing step, the phases were separated by centrifugation (5 min, 10,000 rpm, 4°C). 1 mL of the polar phase was transferred in a glass vial and dried in a vacuum concentrator overnight. The extracellular samples were prepared using 10 μL of the supernatant of the harvested samples and supplementing 500 μL ethanol mixed with 4 μg/mL ribitol as internal standard. The samples were dried under vacuum as well.

Gas chromatography/mass spectrometry measurement was performed after derivatization using a two-step derivatization protocol. Methoxymation was achieved using a methoxyamine hydrochloride solution with a concentration of 20 mg/mL in pyridine followed by silylation applying *N*-methyl-*N*-(trimethylsilyl)-trifluoroacetamide (MSTFA) (Zech et al., 2009; Reimer et al., 2014; Neumann-Schaal et al., 2015). For absolute quantification of 2-hydroxyisocaproate, 3-phenyllactate, 3-phenylpropanoate and phenylacetate a standard-addition was performed. 10 μL of the sample supernatant was supplemented with 10 μL standards-solution between 50 and 400 μM. The samples were mixed with 500 μL ethanol containing 4 μg/mL ribitol as internal standard. Further sample preparation, derivatization and measurement were performed as described above.

#### Gas Chromatography/Mass Spectrometry Based Analysis of Volatile Compounds

Four hundred μL of the culture supernatant were mixed with 60 μL of a HPLC-grade sulfuric acid solution and 600 μL of an internal standard solution of o-cresol. The volatile compounds were extracted by vigorously mixing with 200 μL of *tert*-methylbutyl ether. The ether phase was transferred into a GC-MS vial after centrifugation (5 min, 13,000 rpm, 4°C). The compounds were measured on a Agilent VF-WAXms column (0.25 mm × 30 m, Agilent, Santa Clara, CA, United States) on a Thermo DSQ II gas chromatograph equipped with a liner and quadrupol mass spectrometer as described before (Neumann-Schaal et al., 2015). For quantitative analysis of isocaproate and isovalerate an external calibration was performed. Samples were prepared from a 100 mM solution of both compounds. For calibration solutions between 10 μM and 7 mM were used. Calibration solutions and samples were prepared and measured equivalent to the samples described above.

#### Liquid Chromatography/Mass Spectrometry Based Analysis of Coenzyme A Derivatives

For the analysis of CoA derivatives frozen precipitated cells were extracted in methanol with a concentration of 7 mg/mL. Cells were lysed at -10°C using a Precellys 24 system (Peqlab, Erlangen, Germany) in three cycles of homogenization (6,800 rpm, 30 s with equivalent breaks). The lysate was mixed with 10 mL ice cold ammonium acetate (25 mM, pH 6) and centrifuged (5 min, 10,000 rpm, 4°C). CoA derivatives were extracted using a Strata XL-AW solid phase column (Phenomenex, Aschaffenburg, Germany) as described previously (Wolf et al., 2016).

The CoA derivatives were measured on a Dionex ultimate 3000 system (Thermo Scientific Inc., Waltham, MA, United States) coupled to a Bruker MicroTOF QII mass spectrometer (Bruker Daltonic GmbH, Bremen, Germany) equipped with an electrospray interface. The separation was carried out on a C_18_ analytical column (Gemini 2.0 × 150 mm, particle size 3 μm; Phenomenex) and the data analysis was done as described previously (Wolf et al., 2016).

#### High Performance Liquid Chromatography Based Determination of Amino Acids

High performance liquid chromatography measurements of free amino acids were performed on a 1260 Infinity HPLC system equipped with a fluorescence detector (Agilent Technologies, Waldbronn, Germany) and a Poroshell HPH-C18 separation column (4.6 mm × 100 mm, particle size 2.7 mm; Agilent Technologies). Before measuring, ammonium was precipitated from the samples by a 1:1 dilution with sodium tetraphenylborate (250 mM). After vigorously mixing and centrifugation (5 min, 13,000 rpm, 4°C), the ammonium-free samples were transferred in HPLC vials. The HPLC method was used as described previously (Trautwein et al., 2016; Dannheim et al., 2017b) with the minor modification that mobile phase A was changed to 25 mM sodium acetate (pH 6.5).

#### Data Analysis

Data processing of intracellular metabolites was performed as described previously (Neumann-Schaal et al., 2015; Dannheim et al., 2017a). After processing of the raw data from the GC-MS measurements with the in-house software MetaboliteDetector (version 2.2 N-2013-01-15; Hiller et al., 2009), peak identification was performed non-targeted with a combined compound library. The peak areas of the detected metabolites were normalized to the corresponding internal standard (o-cresol or ribitol) and derivatives were summarized.

### Toxin Quantification

Quantification of toxin A and B in the supernatant was performed using the TGC-E002-1 ELISA (tgcBIOMICS GmbH, Bingen, Germany) after instructions of the manufacturer for the extracellular samples and after 48 h of cultivation. Culture supernatant was harvested by centrifugation (10 min, 10,000 rpm, 4°C) and sterile filtration. The samples were analyzed immediately or after short storage at 4°C.

### Enzymatic Monitoring of Serine Biosynthesis in Crude Cell Extracts

The enzymatic assays using crude extracts were performed as follows: Cells were grown to the early exponential and early stationary phase, harvested by centrifugation and re-suspended in 50 mM HEPES (pH 7.0) supplemented with 1 mg/mL lysozyme and incubated on ice for 60 min. Cells were disrupted by sonication twice with 2 min pulse and 2 min of cooling. The enzymatic assay was performed with 10 μL of crude extract in a total volume of 1 mL in 50 mM HEPES buffer (pH 7.0) and 1.8 mM NADP^+^ or NAD^+^ and reduced by the addition of sodium dithionite. After stabilization of the absorption at 340 nm for at least 2 min, the reaction was started by the addition of 1.5 mM 3-phosphoglyceric acid and absorption increase was followed at 340 nm and 30°C over time. To follow the formation of serine, we used the identical experimental setup, but started the reaction by the addition of 1.5 mM 3-phosphoglyceric acid, incubated for 30 min at 22°C and finally stopped by the addition of 250 μL chloroform. Samples were vigorously mixed for 5 min and centrifuged at 13,000 rpm and 4°C for 10 min. The polar phase was subsequently analyzed for its amino acid content.

## Results

### Adaptation During the Transition From the Growing to Non-growing State

*C. difficile* 630Δ*erm* was grown anaerobically in defined medium. Five samples were taken along the cultivation process to follow the molecular adaptation at the transcriptome, proteome and metabolome level. Samples were taken from the logarithmic growth (exp), the transition phase between growing and non-growing cells (trans), at the point where bacterial growth stopped (stat1), 5 h (stat2) and 10 h into the stationary phase (stat3).

**FIGURE 1:**
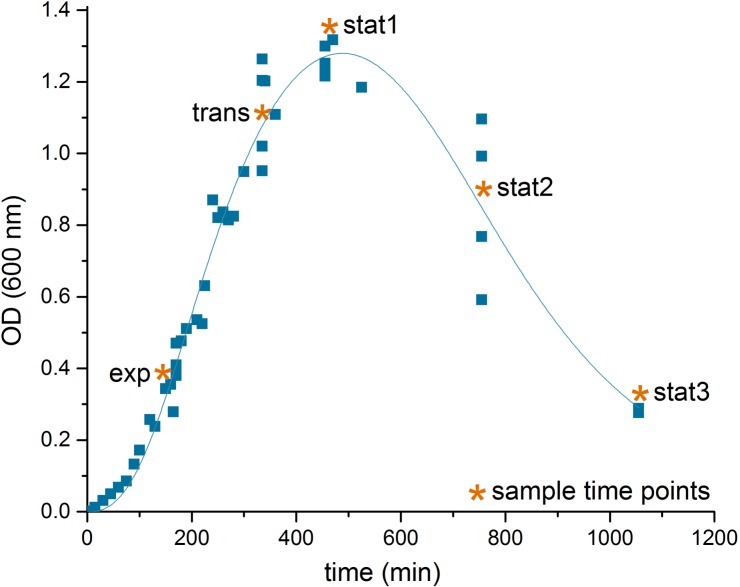
Growth curve of *C. difficile* 630Δ*erm.* Shown is the growth curve of *C. difficile* 630Δ*erm* in casamino acids medium of four biological replicates, lag phase corrected, and the sampling time points (^∗^) in the exponential phase (exp), the transient phase (trans), and three times in the stationary phase (stat1-3). The curve was fitted according to the biphasic Hill Equation using OriginPro, 2016 software (OriginLab Corporation, MA, USA); reduction of the optical density after time point stat1 was induced by aggregation of the cells in the culture medium.

The transition was accompanied by marked changes in the OD and an increase in cell aggregation observed during sampling (**Figure 1**). As observed for other bacteria, the transition from a growing to a non-growing state was accompanied by major transcriptional rearrangements (Jozefczuk et al., 2010; Blom et al., 2011). In our study, the transcriptome data based on DNA microarrays revealed significant changes for 1,439 transcripts over the five time points sampled with the exponential phase samples as reference (*p*-value < 0.05, **Supplementary File S1**). The number of genes whose expression was regulated increased from transition to late stationary phase (stat3, **Table 1**).

**Table 1:** Overview of all systematic levels.

	Transcriptomics		Proteomics		Metabolomics
			
	Upregulated genes	Downregulated genes	Proteins with increased amount	Proteins with decreased amount	Detected metabolites
Exp	–	–	–	–	133
Trans	126	90	431	243	140
Stat1	157	100	457	259	132
Stat2	308	134	778	544	126
Stat3	318	206	900	584	120


*Genes and proteins with -1 < log2 FC > 1 of the transient phase (17.25 h, trans) and the stationary phase (19.25: stat1, 24.25: stat2, 29.25 h: stat3) compared to samples from the exponential phase 14.5 h (exp) as reference state were designated to be regulated expressed/produced. As detected metabolites the extracellular as well as the intracellular detected metabolites were summarized.*

Complementary to the results obtained for the transcriptomic data, we performed an *indepth* proteome analysis for the five time points of interest. Three different subcellular fractions (cytosolic proteins, membrane fraction and proteins secreted into the growth medium) were analyzed (**Supplementary Figure S2** and **Supplementary File S2**). With 3,781 open reading frames predicted in *C. difficile*, we were able to identify overall 55% of the theoretical proteome (**Table 2**). When compared to other systems biology studies on closely related Gram-positive bacteria, both the quality of the fractionation approach as well as the coverage of the proteome was at the upper level and represented the most complete protein inventory of *C. difficile* 630Δ*erm* so far (Becher et al., 2009; Otto et al., 2010).

**Table 2:** Overview of proteomic subfractions.

Predicted localization	Cytosol	EMF	Extracellular	Combined	Theoretical proteome	% of theoretical
Cytoplasmic	963	1184	643	1314	2063	64%
Cytoplasmic membrane	119	394	60	402	942	43%
Unknown	153	238	132	288	688	42%
Cell wall	26	31	35	39	45	87%
Extracellular	5	10	14	20	43	47%
All Proteins	1266	1857	884	2063	3781	55%


*Number of proteins identified for every proteomic subfraction analyzed with assignment to the respective predicted localization. The coverage of the theoretical proteome refers to the percentage of identified proteins in all subfractions combined for a specific predicted localization compared with the theoretical proteome*.

In the metabolic study, a comprehensive extraction and analysis of the intracellular and extracellular metabolites by gas chromatography (GC) and HPLC combined with mass-spectrometry was performed. Overall 90 of 122 detected compounds (**Supplementary File S3**) were assigned to known metabolites based on mass spectra and retention index. In addition, a total of 29 CoA derivatives were identified for all growth phases. The amino acid content of the culture supernatant was quantified by HPLC. Here, 16 out of the 20 proteinogenic amino acids were analyzed together with ornithine and 5-aminovalerate, the reductive Stickland product of proline. As other, we observed clear cut differences between the transcriptome and proteome data. These are due to the poorly understood processes of mRNA stability and controlled RNA degradation, translational control at the ribosome, protein modification and degradation (Hanson and Coller, 2018; Manzoni et al., 2018). Moreover, transcriptomes and proteomes from samples collected at identical time points were compared. However, a certain delay between transcription and translation has to be taken into account (Gunawardana and Niranjan, 2013).

### General Adaptation to Non-growing Conditions

**FIGURE 2:**
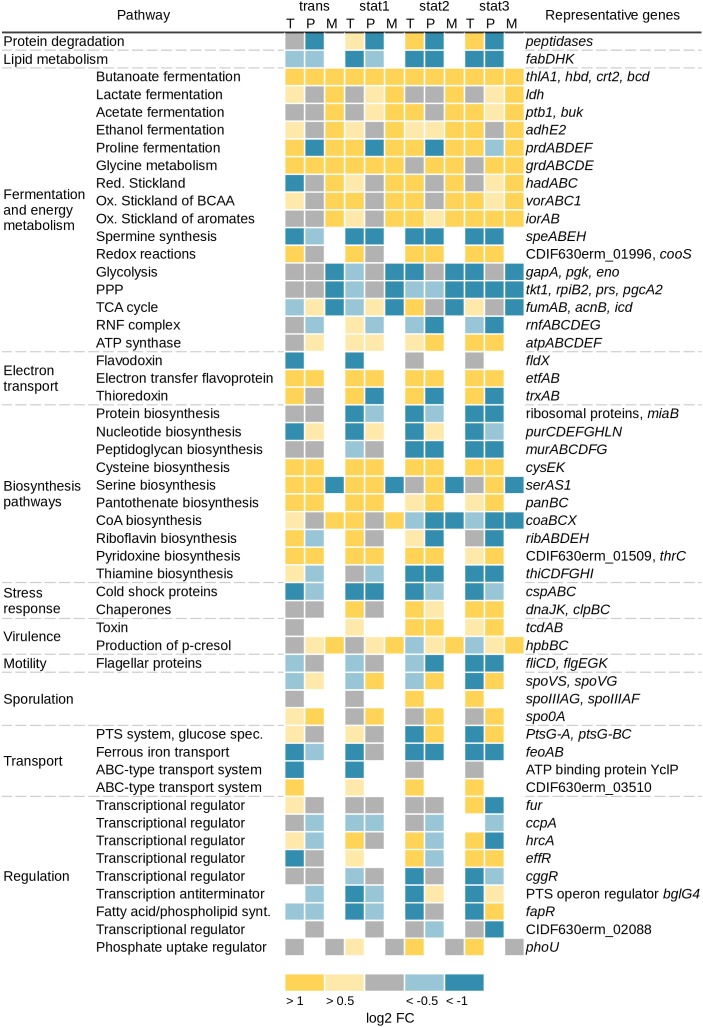
Overview of changed pathways on transcriptomic (T), proteomic (P), and metabolic (M) level. Shown are the log2 fold changes of the transcriptomic and the proteomic data from the sampling time points trans, stat1, stat2, and stat3 with the samples of the exponential phase as reference. Yellow squares represent an increase of gene expression/protein production with log2 FC 0.5–1 for the light color and >1 for the dark color. Blue squares represent a decrease of gene expression/protein production with log2 FC –0.5 to –1 for the light color and <–1 for the dark color. Gray squares represent no changes with log2 FC between –0.5 and 0.5, white squares: not detected. Metabolite values represent a trend compared to the samples of the exponential phase. Toxin A and B in the proteomic data were detected with an ELISA (see section “Materials and Methods”), TCA, tricarboxylic acid; BCAA, branched chain amino acids; PPP, pentose phosphate pathway; ox, oxidative; red, reductive.

Major alterations were seen in the transcriptomic and especially in the extracellular proteomic data for genes and corresponding proteins involved in motility (*fliCD* and *flgEGK*), which were found up to 101-fold downregulated. Further major changes that could be detected on all systematic levels were found in energy producing and different biosynthesis pathways (**Figure 2**). Primarily, the Stickland reactions and fermentation processes showed increased values up to 31-fold at the transcriptome and up to 15-fold at the proteomic level over the course of growth. In contrast, the genes in the glycolysis, the TCA cycle and the PPP showed only minor regulation over the whole growth curve or were downregulated up to 12-fold at the transcriptome level. In agreement, the detectable corresponding metabolites were less abundant in the stationary growth phase. Also in the stationary phase, expression of genes encoding ribosomal proteins, proteins involved in the lipid metabolism (e.g., *fabDHK*) and proteins annotated as cold shock chaperones (e.g., *cspABC*) were decreased (**Figure 2**). Corresponding transcriptome data revealed a reduced expression up to sixfold. In agreement, proteome data showed a reduction up to fourfold. Also expression of genes involved in the spermine synthesis (e.g., *speABEH*) was found 8-fold reduced at the transcriptional and 10-fold at the proteomics level. Spermine in pathogenic bacteria is synthesized from methionine and involved in central processes including biofilm formation, escape from phagolysosomes, bacteriocin production, toxin activity and protection from oxidative and acid stress (Shah and Swiatlo, 2008). Expression of genes annotated as chaperones (e.g., *dnaJK, clpBC*) and proteins involved in the folding and assembly of proteins were induced up to 21-fold in the transcriptome and up to 3-fold in the proteome in the stationary phase. This is in accordance with other *C. difficile* transcriptomic and proteomic studies over different growth phases (Saujet et al., 2011; Janoir et al., 2013) or about stress response (Jain et al., 2011). Analysis of transcriptomic data for genes usually termed as transition phase markers, including *spo0A, sigH*, and *spoIIAA* (Saujet et al., 2011), showed no significant changes. The cytosolic proteome showed a significant increase only for Spo0A until the transient phase. But in the membrane fraction of the proteomic approach a sporulation initiation inhibitor protein (Soj) became abundant in the stationary phase. The exact role in sporulation of these proteins has not been confirmed for *C. difficile* so far. The regulation of sporulation factors can be correlated to the fact that strain 630Δ*erm* does not sporulate efficiently in CDMM in the analyzed time frame and beyond (up to 48 h, data not shown). Overall, there was almost no significant regulation of the expression of genes involved in the sporulation observed in the transcriptomic data.

### Changes in Stickland Reactions

**FIGURE 3:**
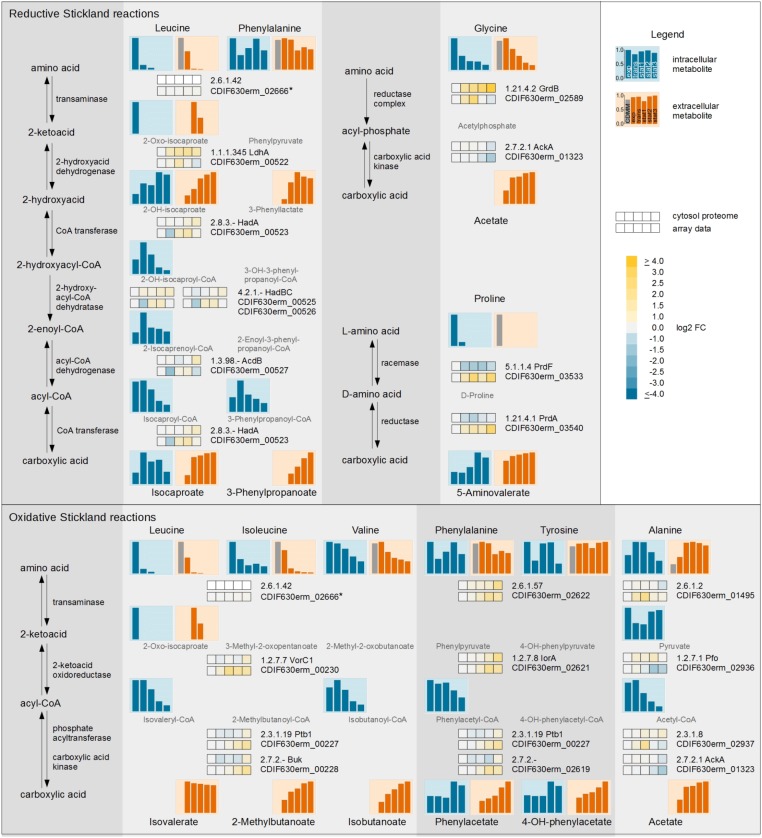
Time resolved systematic overview of reductive and oxidative Stickland reactions in *C. difficile*. On the left side the principal metabolic pathways are depicted. On the right, the individual metabolic pathways of the various amino acids of the reductive **(Upper)** and oxidative **(Lower)** Stickland reactions with the corresponding transcriptomic and proteomic data of representative genes/proteins are shown. The bars and the squares point out the sample time points exp, trans, stat1, stat2, and stat3 from left to right. The squares shows the log2 FC of each time point in comparison with the exponential phase of the cytosolic proteome **(Upper row)** and the transcriptome **(Lower row)**. The bars represent the relative abundance of the metabolites based on the highest concentration; blue: intracellular compounds, orange: extracellular compounds, gray bar in exometabolome: initial concentration in the medium. Missing intermediates were not detected in the GC-MS or LC-MS analysis. ^∗^ not detected in proteomic approach.

Overall, the metabolic pathways of the Stickland reactions can be followed over all three systematic levels and finally integrated into one response (**Figure 3**). The BCAAs (leucine, isoleucine, and valine) as well as phenylalanine, tyrosine, and alanine and the corresponding Stickland intermediates (2-oxo-isocaproate, isovaleryl-CoA, isobutanoyl-CoA, phenylacetyl-CoA, pyruvate and acetyl-CoA) and products (isovalerate, 2-methylbutanoate, isobutanoate, phenylacetate, 4-hydroxyphenylacetate and acetate) were detected for the oxidative Stickland reactions. For the reductive pathway leucine, phenylalanine, glycine and proline and their Stickland products (isocaproate, 3-phenylpropanoate, acetate and 5-aminovalerate) were observed.

Leucine, one of the amino acids used in the reductive, as well as the oxidative pathway of Stickland, was completely consumed in the beginning of the stationary phase. The intermediate 2-oxo-isocaproate of both pathways was detected extracellularly in the transient and early stationary phase. Isovalerate, the product of the oxidative path, showed only minor changes over all time points while isocaproate, the product of the reductive path, was detected in minor quantities in the exponential phase but accumulated intensely in the transient phase. In the stationary phase minor changes were observed due to the depletion of leucine in the medium. The Stickland products of leucine accumulated in the supernatant (68.0% isocaproate and 23.3% isovalerate based on the total leucine content). The (R)-2-hydroxyisocaproate dehydrogenase (LdhA, Kim et al., 2006) was the enzyme with the highest upregulation up to 3.4-fold in the complete leucine degradation pathway over all time points. All other enzymes in the reductive path of Stickland as well as the enzymes in the oxidative path showed only minor changes in the proteomic data. In the transcriptomic approach, predominantly the expression of genes of the oxidative path (*vorC1, iorA, ptb1, buk*) was slightly increased in the late stationary phases.

In the oxidative Stickland reactions, the BCAAs showed the most active conversion. All BCAAs were degraded by the same set of enzymes. In the proteome data, only minor changes of VorC1 as well as Ptb1 and Buk production were detected for the analyzed time frame. Isoleucine was almost completely used up. At the beginning of the stationary phase only about 6.6% were left. At this time point, valine was only consumed up to 48.2%.

Besides leucine, phenylalanine is the second amino acid used in the oxidative as well as the reductive Stickland reactions but it showed only minor assimilation. At the last sampling point, 68.3% of the amino acid was still found in the growth medium. Its oxidative product phenylacetate showed similar levels from the exponential to early stationary phase. It accumulated intra- and extracellularly only in the late stationary phase. In agreement, proteome and DNA array data showed only minor changes before the early stationary phase, while all gene products were found more abundant in the late stationary phases. In the reductive path phenylalanine utilization shares all enzymes with the leucine degradation. The product of this pathway, 3-phenylpropanoate, showed delayed accumulation in the supernatant compared to isocaproate, the product of leucine. 3-Phenylpropanoate was first detected in the transient phase and accumulated constantly up to the late stationary phase. At the end of the cultivation, a concentration of 14.5% 3-phenylpropanoate and only 7.1% phenylacetate in relation to the initial concentration of phenylalanine in the medium were measured.

The other two important amino acids, glycine and proline, are used by *C. difficile* in modified reductive pathways. L-proline is isomerized to D-proline and subsequently reduced to 5-aminovalerate. It is the only Stickland substrate which is not deaminated during the first reaction step. Proline was the amino acid which was consumed the fastest during the reductive Stickland reaction. It was already taken up completely at the beginning of the exponential phase and was fully consumed within the transient phase. The product 5-aminovalerate was exported into the growth medium primarily before the transient phase. Consequently, the proline racemase gene (*prdF*) was downregulated in the transient and stationary phases. Glycine was utilized over the complete cultivation period. Interestingly, the intracellular glycine level peaked in the exponential phase and decreased rapidly until the transient phase, although there was over 70% left in the growth medium. The product acetate accumulated mainly before the transient phase. The proteome data showed constant upregulation of the glycine reductase complex (GrdABDE) over the whole cultivation, while the expression of the corresponding genes was reduced in the late stationary phase (1.5-fold).

### Detection of a Serine Producing Pathway in *C. difficile*

**FIGURE 4:**
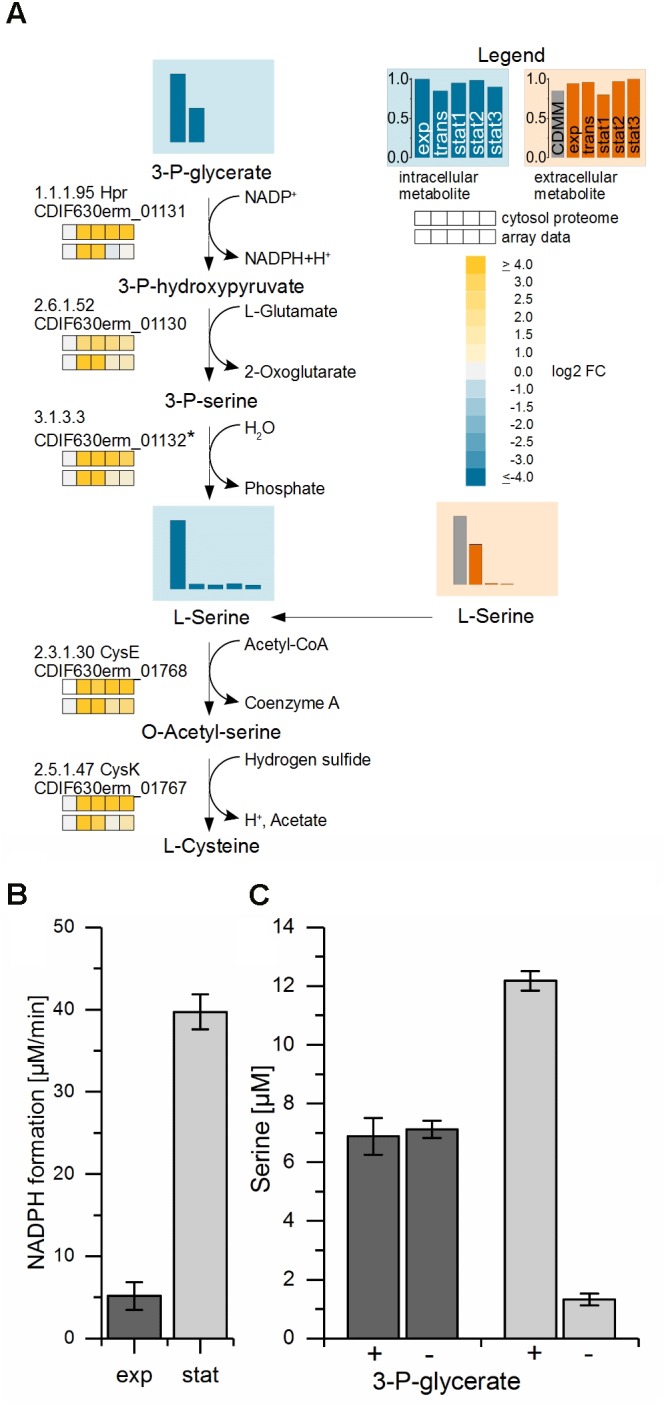
Proposed biosynthesis-pathway of *C. difficile* from the glycolysis substrate 3-phosphoglycerate to serine and cysteine. **(A)** Shown is the proposed pathway from 3-phosphoglycerate via serine to cysteine with the detected metabolite levels of 3-phosphoglycerate (3-*P*-glycerate) and the intracellular and extracellular L-serine values and the corresponding transcriptomic and proteomic data. The bars and the squares point out the sample time points exp, trans, stat1, stat2, and stat3 from left to right. The vertical axis shows the relative abundance based on the highest concentration; blue: intracellular compounds, orange: extracellular compounds, gray bar in exometabolome: initial concentration in the medium. Missing intermediate metabolites were not detected in the GC-MS or LC-MS analysis. The proteomic data of the cytosolic fraction (top squares) and the transcriptomic data (lower squares) show the log2 FC of each time point in comparison with the exponential phase. ^∗^We proposed CDIF630erm_01132, a protein of unknown function located in the same operon with weak homologies to hydrolases, as a candidate for the generation of serine by the removal of the phosphate. **(B)** NADPH formation in the enzyme assay of crude extracts from exponential (dark gray) and stationary phase (light gray) performed at 30°C in a photometer. **(C)** Serine content in the enzyme assay detected in samples of crude extracts from exponential (dark gray) and stationary phase (light gray) analyzed by HPLC with (+) and without (–) the substrate 3-*P*-glycerate performed at 22°C.

The strongest upregulated genes (up to 45-fold) in the stationary phase compared to the exponential phase were the three contiguous open reading frames CDIF630erm_01130-01132 up to 45-fold (**Figure 4A**). These genes are found widespread among Clostridiaceae. The corresponding enzymes were also present at high levels over the whole cultivation time in the proteomic approach. CDIF630erm_01131 is annotated to encode a hydroxypyruvate reductase (Hpr), an enzyme which catalyzes the reversible reaction from D-glycerate to hydroxypyruvate with NADP^+^ as cofactor in the serine metabolism (Dannheim et al., 2017a). CDIF630erm_01130 is annotated to encode a putative serine-pyruvate aminotransaminase and BLAST searches revealed homologies to an alanine-glyoxylate aminotransferase family protein. CDIF630erm_01132 is a protein of unknown function. Our analysis of the amino acid content in the medium showed an almost complete depletion of serine in the stationary phase. In the transient phase only 1.7% of the initial content of serine was detectable in the culture supernatant. Only small levels of glycerate were detected during the whole cultivation process intracellularly (**Supplementary File S3**). Hydroxypyruvate was not detectable at all.

The second highest increase of transcription (up to 32-fold) in the transient phase was observed for the expression of three genes most likely forming an operon (CDIF630erm_01767-01769). The first two of them were annotated to encode a serine acetyltransferase (CysE) and a cysteine synthase (CysK), two proteins catalyzing two consecutive steps of the conversion from serine to cysteine. The third was annotated to encode a ferredoxin. Most likely, it mediates the electron transfer required for this reaction. Cysteine is besides proline the amino acid taken up and degraded first by *C. difficile*.

From these observations we concluded that the enzymes mentioned first including the hydroxypyruvate reductase are used by *C. difficile* in the biosynthetic mode for the production of serine in order to finally synthesize cysteine in a second step. To prove our prediction we analyzed the corresponding enzymatic activities in cell free crude extracts prepared from *C. difficile* cultures grown into the exponential and early stationary phase. For this purpose the cell free extracts from cultures in the exponential and stationary phases were tested with 3-phosphoglycerate as substrate and NADP^+^ as cofactor. We observed a 5–6 times higher NADPH production in assays with crude cell free extracts from bacteria grown into the stationary phase (**Figure 4B**). Additionally, significant *de novo* serine synthesis was observed by HPLC analysis of stationary phase samples. In contrast, in crude cell free extracts obtained from cultures in the exponential phase the serine content remained at an average level (**Figure 4C**).

### Changes in Central Carbon Metabolism

**FIGURE 5:**
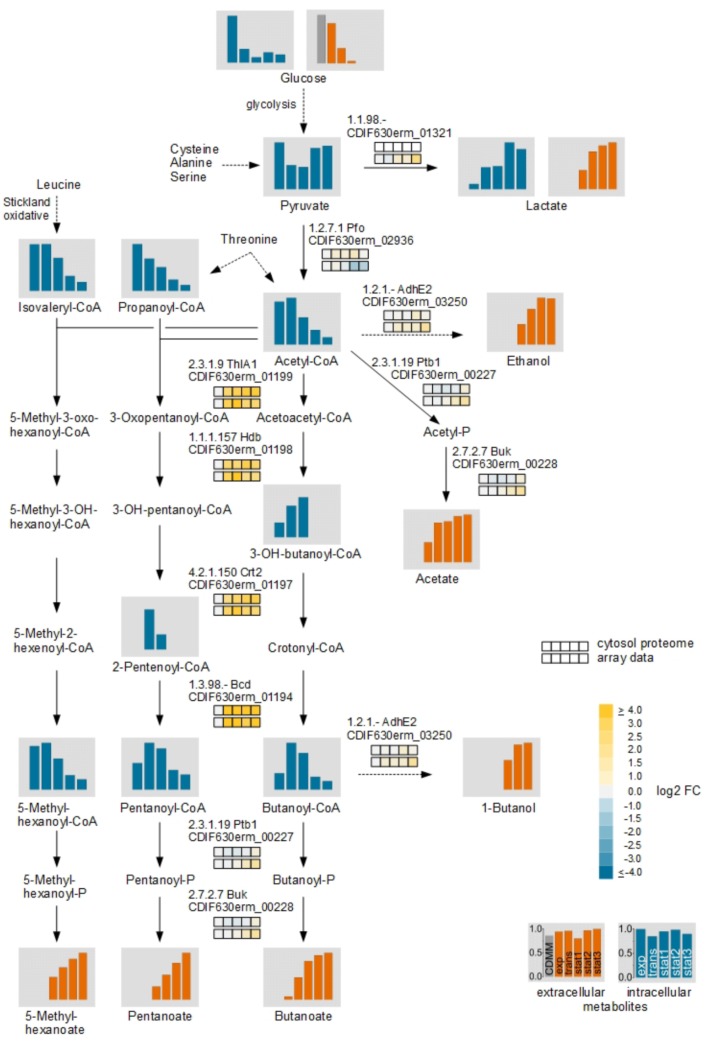
Time resolved analysis of central carbon metabolism of glucose via fermentation by *C. difficile*. The different fermentation pathways from glucose via pyruvate with the detected intra- and extracellular metabolites and the corresponding transcriptomic and proteomic data of representative genes/proteins are shown. The bars and the squares point out the sample time points exp, trans, stat1, stat2, and stat3 from left to right. The squares shows the log2 FC of each time point in comparison with the exponential phase of the cytosolic proteome **(Upper row)** and the transcriptome **(Lower row)**. The bars represent the relative abundance based of the metabolites based on the highest concentration; blue: intracellular compounds, orange: extracellular compounds, gray bar in exometabolome: initial concentration in the medium. Missing intermediate metabolites were not detected in the GC-MS or LC-MS analysis.

During transition to the stationary phase we detected many changes in the central carbon metabolism. In addition to Stickland reactions *C. difficile* uses various fermentation processes linked to the central carbon metabolism to produce energy. Beside glucose, several amino acids like cysteine, alanine, and serine are degraded in the central carbon metabolism via pyruvate. Glycine and threonine degradation enters the central carbon metabolism at the stage of acetyl-CoA. For these derived fermentation pathways significant changes in the metabolite levels were detected (**Figure 5**).

In contrast, the transcriptomic and the proteomic analyses revealed mostly moderate regulation of corresponding genes and enzymes. Only for the pathway from acetyl-CoA to butanoyl-CoA up to 31-fold increased levels of transcripts of the genes encoding the acetyl-CoA acetyltransferase (*thlA1*), 3-hydroxybutanoyl-CoA dehydrogenase (*hdb*), short-chain-enoyl-CoA hydratase (*crt2*) and the butanoyl-CoA dehydrogenase (*bcd*) were detected for all time points after the exponential growth phase. The intracellular detected CoA-derivatives of the butanoate formation pathway showed increased levels in the transient and the early stationary phase. In the late stationary phase the levels decreased and it seemed that the main flux in the central carbon metabolism of pyruvate changed from acetyl-CoA to lactate. In *C. difficile*, two lactate dehydrogenases were annotated. An L-lactate dehydrogenase (Ldh) and an electron bifurcating lactate dehydrogenase (CDIF630erm_01319-01321) (Dannheim et al., 2017a). The Ldh showed only minor regulation in the transcriptomic as well as the proteomic analyses while the transcription of the genes of the electron bifurcating lactate dehydrogenase was found increased up to 2.8-fold in the late stationary phase. In agreement, pyruvate and lactate accumulated in the late stationary phase intracellularly.

### Toxin Detection

**FIGURE 6:**
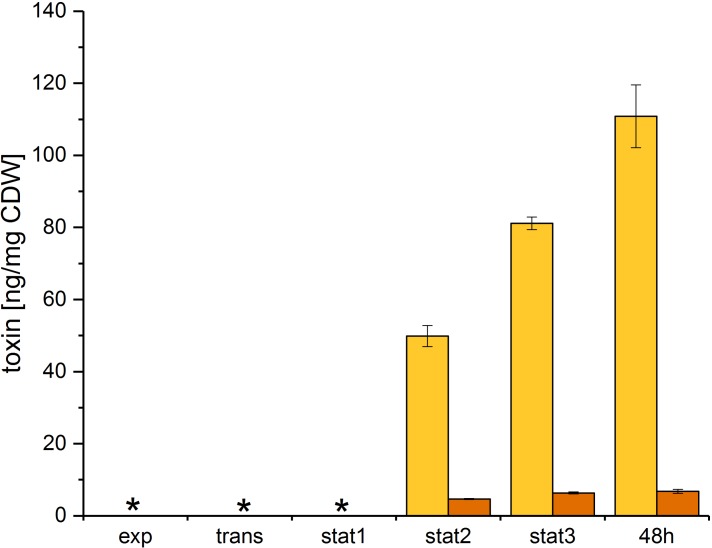
Toxin formation of *C. difficile* 630Δ*erm.* Toxins were quantified at all sample time points discussed above and after 48 h of growth. Toxin A (yellow) and toxin B (orange) were quantified in the culture supernatant using an immunoassay and were calculated per mg of *C. difficile* dry weight; ^∗^ no toxin detectable.

Toxin A and B were quantified using an ELISA assay for the simultaneous detection of both toxins. The toxin levels were determined at all five time points and after 48 h of growth. Both toxins were exported in the stationary phase (**Figure 6**) and accumulated in the culture supernatant. Our data showed a different accumulation for both toxins over the growth curve. Toxin A was exported in the early stationary phase and accumulated in the supernatant up to the 48 h sample time point. Toxin B was also exported in the early stationary phase, but showed only minor accumulation in the late stationary phase.

## Discussion

### Continuous Alterations of Substrate Availability Over the Growth Phases Cause Complex Changes in Stickland Fermentation

Stickland reactions are the dominant energy producing reactions in *C. difficile* (**Figure 3**). The bacterium favors the amino acids proline and leucine for these reactions. However, they were completely consumed before the onset of the stationary phase. Leucine showed a shift from the oxidative to the reductive Stickland pathway (isovalerate and isocaproate as products) between the exponential and the transient growth phase in accordance with earlier findings (Neumann-Schaal et al., 2015).

The other mainly used amino acids were isoleucine, glycine and valine. Isoleucine was also almost completely consumed in the stationary phase. There were only about 3% left at the end of the cultivation. The end product of the fermentation, 2-methylbutanoate, was exported into the growth medium during the whole cultivation process. The valine level in the culture supernatant was at about 52% of the initial concentration at the beginning of the stationary phase and remained at 40% at the end of the cultivation. In contrast, its fermentation product isobutanoate was found secreted over the whole time. In accordance, the expression of the genes *vorABC, ptb123*, and *buk* involved in the oxidative Stickland degradation of BCAA was increased in the late stationary phase. However, the residual intracellular levels of isoleucine and valine in combination with the lower product formation and the increase of the *vorABC, ptb123*, and *buk* gene expression pointed toward a lack of reduced ferredoxin or CoA for the reductive formation of acyl-CoA. The decline of the intracellular isobutanoyl-CoA level and the decrease of intracellular free CoA in the stationary phase (**Supplementary File S3**) confirmed this assumption. Analogously, the biosynthesis of pantothenate, which is used for the CoA biosynthesis, was found induced over the whole growth curve, although it was additionally present in the growth medium. In the membrane fraction of the proteomic approach, a putative pantothenate transporter (PanT) showed high abundance at all sample time points reaching its maximum in the early stationary phase (stat1). Pantothenate is a precursor of CoA, which is used in the Stickland reactions as well as the central carbon metabolism (Miller and Schlesinger, 1993). The biosynthesis of CoA was induced before the onset of the stationary phase. Finally, free intracellular CoA decreased in the late stationary phase which may be linked to a lack of cysteine or pantothenate required for CoA biosynthesis (**Supplementary Figure S3**).

Glycine was the only amino acid, which showed high consumption in the stationary growth. At the beginning of the stationary phase, about 48% of the initial glycine content could be detected and at the end of the cultivation only 16% were left. Previous studies about the regulation of the expression of glycine reductase (*grd*) and the D-proline reductase (*prd*) genes showed interesting differences (Jackson et al., 2006; Bouillaut et al., 2013). The addition of proline to the medium increased the proline reductase formation with a simultaneous reduction of glycine reductase levels via the respective gene regulation by the PrdR regulator (Bouillaut et al., 2013). Taking the perceived metabolite levels into account, this observation could explain the higher degradation of glycine in the stationary phase when proline was already metabolized. Anyway, a complex view on all levels of regulation showed that both enzyme complexes were regulated similarly before the onset of the stationary phase on transcriptional level. At the beginning of the stationary phase (stat1) the transcription of the genes for both enzyme complexes (*prdABDEF, grdABDE*) were found upregulated up to sixfold. Subsequently, the transcript levels for the glycine reductase were found somewhat reduced, although there was still glycine left in the medium. However, the proteome showed a strong increase of the glycine reductase complex in the stationary phase. Especially the membrane associated GrdA in the EMF was abundant with a fold change up to 104 in the stationary phase. The discrepancy between transcriptomic and proteomic data for the glycine reductase complex in the late stationary phase can be attributed to the stability of the enzyme complex or to the usual delay of translation versus transcription, as transcriptome and proteome samples were compared at the same time point (Gunawardana and Niranjan, 2013). The transcription of genes of the proline reductase complex increased up to 6.8-fold in the late stationary phase (stat3), although there was no proline left in the medium. In the microarray data of the late stationary phase, the expression of the gene CDIF630erm_02215 showed a similar upregulation of up to 6.5-fold as the proline reductase genes located in close proximity. This gene is annotated as putative proline iminopeptidase (Dannheim et al., 2017a), an enzyme that selectively removes N-terminal proline residues from peptides. A secession of proline from cellular peptides and proteins is in accordance with the renewed high expression of the proline reductase genes in the late stationary phase. However, an increase of free intracellular proline was not detected which may be assigned to a presumed fast degradation. Further, the proportion of the entire product 5-aminovalerate would probably be very low.

The aromatic amino acids, phenylalanine and tyrosine, were metabolized only in minor quantities, but the fermentation products of the oxidative path, phenylacetate and 4-hydroxyphenylacetate, were secreted over the whole growth curve, especially in the late stationary phase. This was consistent with the transcriptomic and proteomic data, where the expression of the genes encoding the aromatic-amino-acid aminotransferase (CDIF630erm_02622) and the indolepyruvate oxidoreductase (*iorAB*), which show a broad substrate specificity toward all aromatic amino acids (Mai and Adams, 1994; Schut et al., 2001), was increased in the late stationary phase. The reductive fermentation product of phenylalanine, 3-phenylpropanoate, showed a similar behavior as those for leucine and is produced by the same set of enzymes (HadABC, LdhA, and AcdB) primarily used for leucine degradation. It was firstly detected in the transient phase in the supernatant while its level increased in the stationary phase.

The two amino acids leucine and phenylalanine were used both in oxidative and reductive Stickland pathways. In the reductive path they were degraded by the same set of enzymes (Neumann-Schaal et al., 2015; Dannheim et al., 2017a). The (R)-2-hydroxyisocaproate dehydrogenase (LdhA) (Kim et al., 2006) showed the highest upregulation of up to 3.4-fold in the complete reductive degradation over all time points in the proteome. All other enzymes in the reductive path for leucine degradation as well as the enzymes in the oxidative path showed only minor regulation. In combination with the upregulation (up to 3.7-fold) of the gene encoding the indolepyruvate oxidoreductase (*iorAB*) in the stationary phase, these findings showed that *C. difficile* uses leucine in casamino acids containing medium first for the oxidative Stickland reaction. But already in the exponential phase, the bacterium switched to the reductive pathway. Phenylalanine seemed to be used in both pathways in the stationary phase. In the reductive path the amount of used leucine was about three times and for phenylalanine two times higher than in the oxidative pathway (**Table 3**).

**Table 3:** Quantification of the Stickland products from leucine and phenylalanine.

	Leucine degradation (%)	Phenylalanine degradation (%)
Oxidative path	23.3	7.1
Reductive path	68.0	14.5


*Their total content in the supernatant at the end of the cultivation (stat3) was analyzed by HPLC-FLD. The amount was calculated from the initial concentration of the corresponding amino acid in the culture medium*.

### Biosynthesis of Serine as a Basis for Cysteine Replenishment

Besides proline, cysteine is the amino acid taken up and degraded first by *C. difficile*. Here, cysteine might be used by the bacterium for biomass production and additionally via degradation to pyruvate for energy generation (Dubois et al., 2016). Cysteine is also known to reduce toxin production in *C. difficile* when present in the growth medium (Karlsson et al., 2000; Dubois et al., 2016).

Consequently, we proposed, that the three enzymes encoded by CDIF630erm_01130-01132 catalyzed the reaction from 3-phosphoglycerate to L-serine: in the first step, the protein encoded by CDIF630erm_01131 acts as 3-phosphoglycerate dehydrogenase and catalyzes the reaction from 3-phosphoglycerate to 3-phosphohydroxypyruvate. In the second step, a phosphoserine transaminase encoded by CDIF630erm_01130 catalyzes the further reaction to 3-phosphoserine with the deamination of glutamate to 2-oxoglutarate. In the last step, L-serine is generated by the removal of the phosphate. A candidate for this reaction is a protein of unknown function encoded in the same operon (CDIF630erm_01132) which shows weak homologies to hydrolases. In a next step, the serine acetyltransferase and the cysteine synthase likely synthesize cysteine from the formed serine (**Figure 4A**). This way, *C. difficile* can use the glycolysis in the late exponential to transient phase, when the favored amino acids for Stickland reactions are depleted, to produce serine as a precursor of cysteine.

A previous transcriptomic approach showed the influence of cysteine on the transcription of genes involved in amino acid biosynthesis, fermentation, energy metabolism, iron acquisition and stress response (Dubois et al., 2016). In our analyses, the expression of genes involved in the reaction from cysteine to pyruvate revealed no obvious regulation over the whole growth curve with the exception of cystathionine beta-lyase gene (*malY)*, whose transcription was decreased up to sixfold in the late stationary phase. In contrast, the expression of genes for thioredoxin (*trxA1*) and a thioredoxin reductase (*trxB1*) were found upregulated up to 10.3-fold in the late stationary phase. The increase of transcripts involved in thiol protection and stress response (Dubois et al., 2016) suggests that cysteine is needed for the survival of *C. difficile* in the late stationary phase. This in turn explains the need for the biosynthesis of this amino acid after its depletion in the culture supernatant.

### Changes in the Central Carbon Metabolism Are Related to the Energy Metabolism and Toxin Production

In the central carbon metabolism glucose was completely consumed at the beginning of the stationary phase and the main pathway changed from glucose catabolism via acetyl-CoA and butanoate fermentation to the reduction of pyruvate to lactate (**Figure 5**). Due to the depletion of preferred reductive Stickland substrates such as proline and leucine, *C. difficile* mainly oxidized Stickland amino acids in the stationary phase while NAD^+^ or ferredoxins seems to be recovered in other pathways. The changes of the metabolic fluxes from the fermentation of amino acids to the central carbon metabolism during adaptation to the non-growing state and its connection to the toxin production suggested a high influence of the metabolism of the host and the available nutrients in the gut on the course of disease. The correlation of the metabolism of *C. difficile* to that of the host was already shown *in vivo*, where the bacterial metabolism alters depending on nutrient availability, the different phases of disease and the bacterial community which in turn is strongly influenced by the use of antibiotics (Jenior et al., 2017; Fletcher et al., 2018).

Fermentation with the L-lactate dehydrogenase resulted in a direct oxidation of one NADH while fermentation with the electron bifurcating lactate dehydrogenase resulted in the oxidation of two reduction equivalents (Weghoff et al., 2015; Buckel and Thauer, 2018). In the butanoate fermentation two reducing equivalents are oxidized. The switch to lactate fermentation could be due to the need of a faster process of consuming reducing equivalents or the limited availability of free CoA (**Supplementary File S3**).

The proteomic approach showed also differences in other compounds for energy transport. In the membrane fraction, the Rnf-complex RnfABDE was decreased up to 7.7-fold in the late stationary phase. With the Rnf-complex electrons from reduced ferredoxin were transferred to one NAD^+^ by generating a sodium ion gradient across the cytoplasmic membrane (Biegel et al., 2011; Tremblay et al., 2012). The produced sodium ion gradient can be used for ATP synthesis. Simultaneously, the energy-coupling factor transporters (EcfA1, EcfA2, and EcfT) were decreased up to 16.9-fold in the late stationary phase on proteome level. The Ecf transporter is used for the import of micronutrients by the hydrolysis of ATP (Zhang, 2013; Swier et al., 2016). Interestingly, the ATP synthase (CDIF630erm_03237-03244) was found to be up to sixfold induced in the late stationary phase in the transcriptomic as well as the proteomic approach in our study. Overall, our data support the complex correlation between the different energy transport systems and the associated import of nutrients, which could be in total related to the toxin production.

It is known that the central carbon metabolism is regulated by glucose itself as well as by different global regulators (Sonenshein, 2005; Dineen et al., 2010; Antunes et al., 2012; Bouillaut et al., 2015). Also certain metabolites show an influence on the regulatory activity based on signaling pathways (Kochanowski et al., 2017). 50% of the genes regulated by glucose are based on the catabolite control protein CcpA which could be a link between the carbon- and the nitrogen pathways (Antunes et al., 2012). In our study, we failed to detect significant regulation of CcpA in our transcriptome and proteome data. There is not much information available on the regulation of CcpA production and stability in Clostridia. Moreover, the regulatory principles of CcpA from Clostridia differ from those of Bacilli (Yang et al., 2017). Very recently some autoregulation of the *ccpA* gene from *C. acetobutylicum* was observed (Zhang et al., 2018). However, the observed degree of regulation did not suggest major concentration changes of the protein in the cell. Other known regulators in the central carbon metabolism to adaptive stress response by nutrient limitation are CodY and Rex. CodY regulates the expression of genes depending on the intracellular BCAA and the GTP content, and Rex regulates the expression of most of the identical genes in response to the NAD^+^/NADH ratio (Sonenshein, 2005; Dineen et al., 2010; Bouillaut et al., 2015). In our study, the expression of both, *codY* and *rex*, showed no significant regulation during the whole cultivation time at the level of the transcriptome and proteome. CodY is also known to repress the butanoate synthesis (Dineen et al., 2010) but this effect was not observed in our study (**Figure 5**).

Both toxins were exported in the early stationary phase and accumulated in the supernatant up to the 48 h sample time point. In contrast to toxin A, toxin B showed only minor additional accumulation in the late stationary phase. This difference could be due to the very different toxin concentrations, which may influence the export efficiency. Previous studies led to contradictory results concerning intracellular toxin levels: early studies showed that in exponential growth, toxin detection is possible neither intracellularly nor in the supernatant (Karlsson et al., 2008). In a previous study, we determined minor intracellular toxin formation in the stationary phase (Neumann-Schaal et al., 2015). In contrast, a recent study showed higher intracellular toxin A levels in complex media, but there was no absolute quantification done (Wydau-Dematteis et al., 2018). However, different media and isolates were used. Due to the accumulation of the toxins in the culture supernatant in the late stationary phase an influence of the growth phase or the growth rate could be assumed. But neither variations of the growth phase nor the growth rate showed direct effects on the toxin production (Karlsson et al., 1999). Consequently, a connection of toxin formation and metabolism in general or certain compounds of the metabolism was supposed. Here, the global regulator Rex (CDIF630erm_00292) could react to shifted ratios of NAD^+^/NADH due to the major rearrangements in the fermentation processes. The NAD^+^/NADH ratio in our study decreased following the course of growth (**Supplementary File S3**). It was shown that Rex is active as a repressor of target genes by high NAD^+^/NADH ratios and dissociates from the operator sites when the NAD^+^/NADH ratio decreases (Brekasis and Paget, 2003; Bouillaut et al., 2015). Also the regulation of the central carbon and the energy metabolism by Rex was shown (Ravcheev et al., 2012). Accordingly, the observed shift in the NAD^+^/NADH ratio could cause the observed regulation of toxin formation in our analysis. Former studies already suggested the link between intracellular NADH, the butanoate metabolism and the toxin formation in *C. difficile* (Neumann-Schaal et al., 2015). Our data support this correlation, and in addition suggested an involvement of the complete energy metabolism, presumably due to the reduction equivalents, and the central carbon metabolism in toxin production. An influence of pyruvate as a product of cysteine degradation on toxin formation was concluded by Dubois et al. (2016). The addition of pyruvate to stationary grown cultures resulted in the repression of toxin genes (Dubois et al., 2016). The increased intracellular pyruvate level in the late stationary phase in our study suggested that less likely the single metabolite was responsible for the repression of toxins but rather a change of the flux in the central carbon metabolism. The more global relationship between the metabolism and the toxin formation is confirmed by the findings that the global regulators CodY and CcpA influence the toxin formation in *C. difficile* (Dupuy and Sonenshein, 1998; Dineen et al., 2010).

Interestingly, expression of genes involved in the biosynthesis of riboflavin (*ribABDEH*) were induced before the onset of stationary phase and showed decreased levels in the late stationary phase when the toxins were secreted. Precursors of riboflavin are known to activate mucosal-associated invariant T cells, which are involved in the immune response in humans (Corbett et al., 2014). This may lead to a limited response of the innate immune system to *C. difficile* while toxins are secreted.

## Conclusion

Using an integrated time-resolved multi-omics approach we were able to unravel the global transcriptional, proteomic and metabolic changes taking place when *C. difficile* is shifting from a growing to a non-growing state. We showed that *C. difficile* has depleted all of its favored amino acids for energy production by Stickland-type fermentative reactions and also cysteine at the end of the exponential phase. This led to drastic rearrangements of the metabolic flux. Previously used amino acids in the oxidative Stickland reactions were then mainly used in the reductive paths. Furthermore, the central carbon metabolism associated fermentation pathways showed higher activity. Most of all, the degradation pathway from acetyl-CoA to butanoate, pentanoate and 5-methylhexanoate were found increased. In the late stationary phases the amounts of the CoA intermediates decreased presumably due to a lack of free CoA. This leads to a further metabolic shift to the fermentation of lactate. In both pathways, NADH is oxidized to NAD^+^. We showed that *C. difficile* is able to replenish serine and thereby most likely also cysteine from 3-phosphoglycerate. Another favored amino acid proline may be selectively removed from peptides or proteins by an iminopeptidase in the late stationary phase.

In the late phases of growth during onset of toxin secretion, the central carbon metabolism showed higher intensities. The correlation between butanoate formation and the toxin production has been suspected in some other studies (Karlsson et al., 2000; Neumann-Schaal et al., 2015). The central role of pyruvate has been discussed before (Dubois et al., 2016). We showed, that there must be a more general correlation between the energy metabolism, most of all, the central carbon metabolism, and the toxin formation in *C. difficile*. Our data indicate the complexity of the processes influencing the toxin production in *C. difficile* based on nutrient availability. There is the need for more detailed analyses, especially focusing on the energy metabolism, to understand the whole system of toxin production.

## Author Contributions

JH, AO, DB, DJ, and MN-S conceived the idea. JH, AO, MB, A-MM, and MN-S performed the experiments. JH, AO, MB, RB, DJ, and MN-S analyzed the data. JH, DJ, and MN-S wrote the manuscript with input of all authors. All authors edited the manuscript and approved the final version.

## Conflict of Interest Statement

The authors declare that the research was conducted in the absence of any commercial or financial relationships that could be construed as a potential conflict of interest.

## Footnotes

1 http://www.cran.r-project.org/

## Abbreviations

BCAA: branched chain amino acids
CDMM: casamino acids medium
CoA: coenzyme A
EMF: enriched membrane fraction
FLD: fluorescence light detector
GC-MS: gas chromatography/mass spectrometry
HPLC: high performance liquid chromatography
LC-MS/MS: liquid chromatography with tandem mass spectrometry
LFQ: label-free quantification
log2 FC: logarithmic fold change with base 2
OD: optical density
Ox: oxidative
PPP: pentose phosphate pathway
Red: reductive
TCA: tricarboxylic acid
