# Supplementary figures and images for: Metabolic Reprogramming of Clostridioides difficile During the Stationary Phase With the Induction of Toxin Production
Source: Front Microbiol. 2018 Aug 21;9:1970. doi: 10.3389/fmicb.2018.01970 (PMC6110889; doi:10.3389/fmicb.2018.01970)

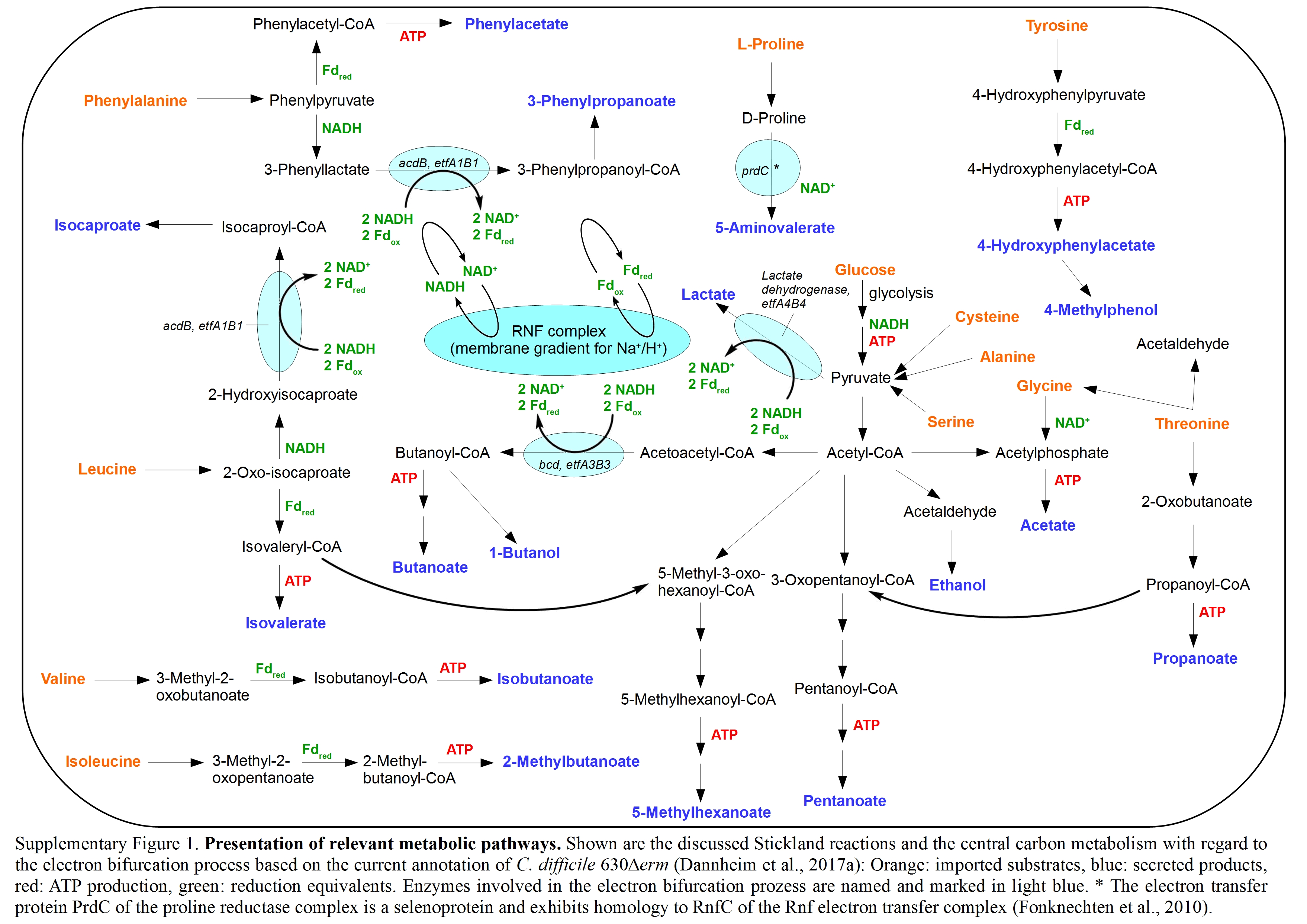

Supplement: Supplementary file 1 [file Image_1.JPEG]

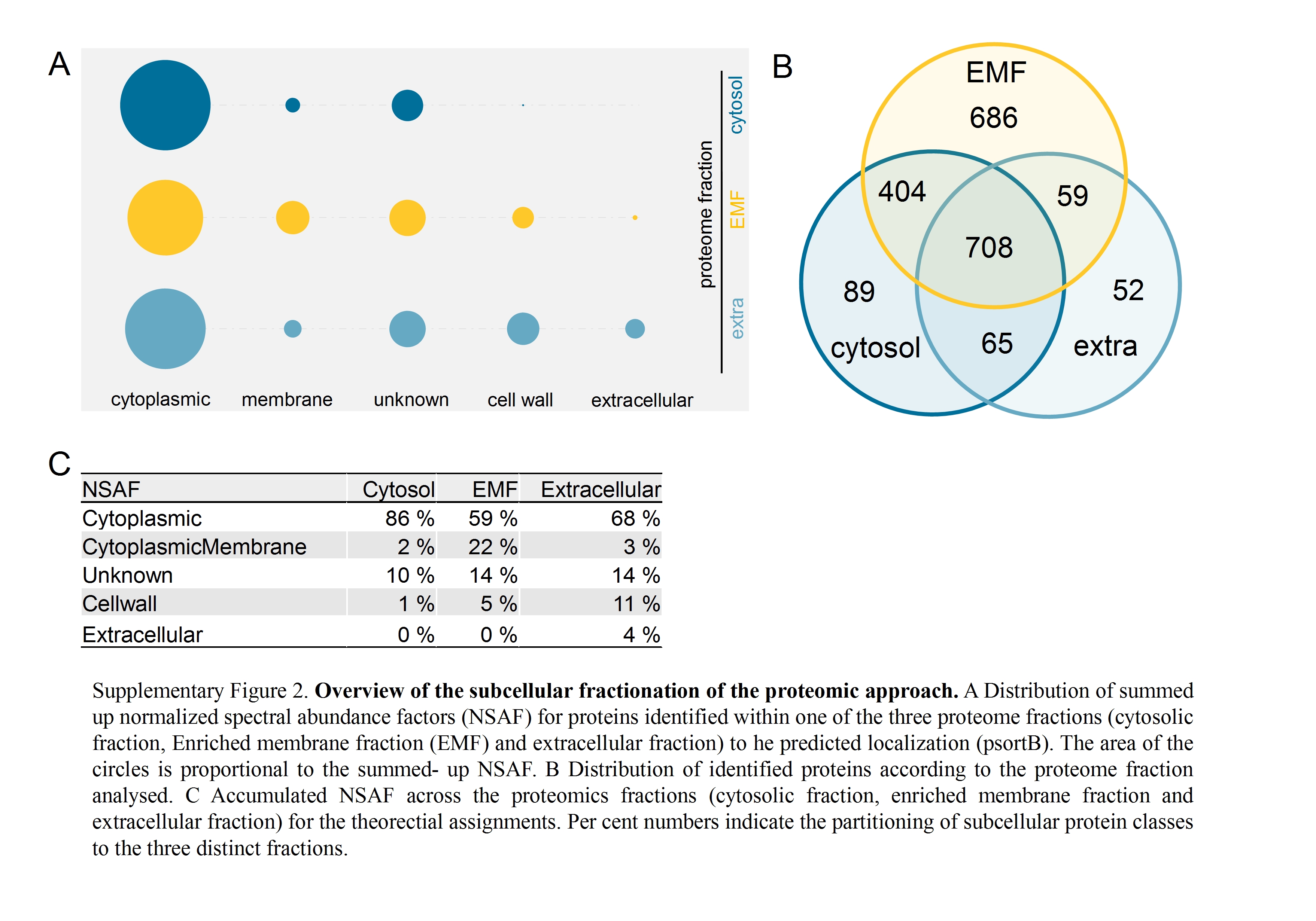

Supplement: Supplementary file 2 [file Image_2.JPEG]

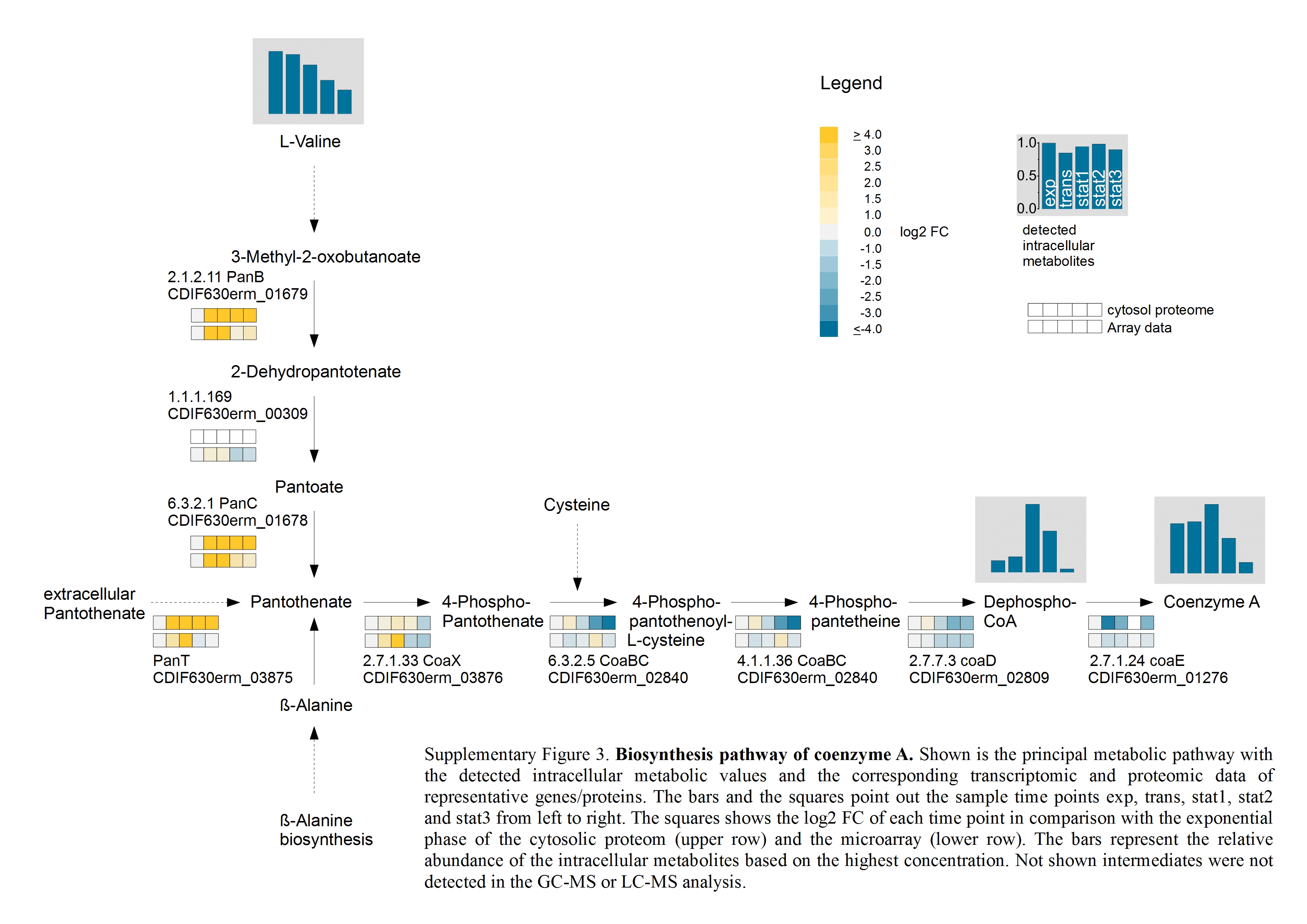

Supplement: Supplementary file 3 [file Image_3.JPEG]
